# A Treatise on Surgery

**Published:** 1906-07

**Authors:** 


**A Treatise on Surgery.** In two volumes. By **George Ryerson Fowler,** M.D., Examiner in Surgery, Board of Medical Examiners of the Regents of the University of the State of New York; Emeritus Professor of Surgery in the New York Polyclinic, etc. Two imperial octavos of 725 pages each, with 888 text illustrations and 4 colored plates, all original. Volume I. Philadelphia and London: W. B. Saunders Company. 1906. (Per set: Cloth, $15.00 net; half-morocco, $17.00 net).

We received the first volume of Dr. Fowler's latest surgical work with pleasure, for rarely is the subject of surgery presented to the reader in a more concise or in better arranged form. His classifications are excellent, especially the chapters upon the various pathological conditions which are found under anatomical divisions, thereby making reference and indexing easy. His introductory chapter is well arranged, each subject being presented in a brief, clear cut, comprehensive manner, giving the essentials necessary for a proper appreciation of the relation of medicine and the individual to the surgeon. It is gratifying in this relation to note that in each instance only one or two simple, practical methods are described, instead of many intricate and difficult ones, thereby saving much needless reading matter so often found in works of the kind.

The first portion of this work embraces wounds, inflammation from the surgeon's standpoint, and surgical fevers in their various phases. Following this, classified anatomically, the skin and subcutaneous tissues, vascular system, nervous system, tendons and muscles, osseous and articular systems, are briefly but excellently dealt with. Then follows a chapter on gunshot wounds, with numerous illustrations from the recent Russian-Japanese war, which in turn is followed by infectious diseases, including both the acute wound diseases and the chronic forms. Next in order is a well illustrated chapter on tumors; and finally, the chapter headed—laboratory aids in surgical diagnosis and prognosis, which is unquestionably one of the most useful and practical ever presented, the fund of information contained in this short space being unexcelled and invaluable.

The second portion of this work covers surgical operations arranged as they occur under the various anatomical divisions. There are two valuable introductory chapters, one on operative technic, the other on anesthetics; after which, operations upon the head, neck and thorax are ably described. It is to be regretted that in the chapter on aseptic technic the mention of the use of rubber gloves is omitted, and instead so much space is devoted to hand sterilisation by various chemicals; that in the chapter on surgical shock the value of adrenalin and morphine were not given a more prominent place; lastly, that no mention of the use of Crile's pneumatic suit and of the reverse Trendelenberg position is made in the chapters on operations of the head and neck (especially goiter), the values of these procedures being now well recognised.

These criticisms, however, are but minor blemishes upon a work of great value from an eminent and typical American surgeon. The medical profession will find in it a large fund of valuable knowledge, arranged to suit the needs of the busy man or the more careful student. It is a credit alike to American surgery and to the author, who was one of its most brilliant exponents. J. P.

